# Concurrent Sigmoid Microperforation in the Setting of Respiratory Syncytial Virus Infection in an Adult Patient: A Case Report

**DOI:** 10.7759/cureus.115710

**Published:** 2026-09-03

**Authors:** Brian P Ireland, Valerie T Bakly, Stephen Howard, Mohammad Jawish

**Affiliations:** 1 Internal Medicine, Lake Erie College of Osteopathic Medicine, Rochester, USA; 2 Internal Medicine, Rochester Regional Health (RRH) Unity Hospital, Rochester, USA; 3 Internal Medicine, Rochester Regional Health, Rochester, USA

**Keywords:** gastrointestinal perforation, intra-abdominal abscess, microperforation, respiratory syncytial virus (rsv), rsv infection, sigmoid perforation

## Abstract

Respiratory syncytial virus (RSV) is a common respiratory pathogen primarily associated with pediatric infections; however, RSV infection is recognized as a significant cause of illness in adults, particularly during winter months. Despite its well-established association with respiratory disease, RSV can also present with extrapulmonary manifestations, including gastrointestinal symptoms. Here, we present the case of a 65-year-old male who presented with dyspnea and abdominal pain and tested positive for RSV. Concurrently, the patient was discovered to have a perforated sigmoid diverticulitis on abdominal imaging. Surgical intervention was deferred due to a high perioperative risk, and the patient was treated conservatively with broad-spectrum antibiotics. Shortly after, the patient developed multiple abdominal abscesses, which were treated with CT-guided percutaneous drainage and drain placement. RSV has been documented to cause gastrointestinal pathology in children by weakening the bowel wall. As RSV was identified in this patient in the setting of gastrointestinal pathology, it is hypothesized that RSV may have contributed to this patient’s gastrointestinal condition.

## Introduction

Respiratory syncytial virus (RSV) is a negative-sense, single-stranded RNA virus that is a major contributor to seasonal respiratory illness, particularly during winter months in temperate climates [1]. The United States Centers for Disease Control (CDC) estimates that roughly 2.1 million children younger than the age of five years are seen in the outpatient setting annually for RSV infection, with approximately 58,000-80,000 children hospitalized each year. Additionally, the CDC estimates that 110,000-180,000 adults older than 50 years are hospitalized annually due to RSV infection [2]. Symptoms of RSV are often non-specific and may include nasal congestion, cough, pharyngitis, headache, and low-grade fever. Severe cases may present with significant cough, wheezing, increased work of breathing, and hypoxia. Additionally, there is some evidence to support that gastrointestinal symptoms such as vomiting and diarrhea can occur in the setting of RSV infection as well; however, it has not been identified in epidemiologic research that bowel perforations are an identified sequela of RSV in the adult population [3].

A bowel perforation is a life-threatening condition that allows for gas, fluid, and intestinal contents to leak into the peritoneal cavity, causing a clinical presentation that can range from mild abdominal discomfort to peritonitis and possible septic shock [4]. In neonates, necrotizing enterocolitis represents a well-recognized cause of bowel perforation [5]. Interestingly, some studies have reported cases of necrotizing enterocolitis associated with RSV infection in infants, suggesting that RSV may contribute to gastrointestinal pathology through inflammatory or hypoxic mechanisms [6].

As mentioned, similar gastrointestinal complications associated with RSV infection have not been described in adults. Here, we present a case of RSV infection occurring concurrently with perforated sigmoid diverticulitis and subsequent intra-abdominal abscess formation in an older adult. This report serves as an observational report of these conditions occurring concurrently and generates the hypothesis that RSV in an adult or geriatric patient with comorbidities such as known diverticulitis may contribute to bowel perforation in this demographic.

## Case presentation

A 65-year-old male with a past medical history of hyperlipidemia, hypertension, insulin-dependent type 2 diabetes mellitus, non-ST-elevation myocardial infarction (NSTEMI) with prior percutaneous intervention to the right coronary artery, supraventricular tachycardia treated with ablation, and a 30-pack-year smoking history presented to the emergency department with shortness of breath, persistent cough with increased yellow sputum production, and diffuse abdominal pain. He endorsed several days of abdominal discomfort associated with nausea and vomiting. On the day of presentation, he developed worsening dyspnea at rest, prompting a call to emergency medical services (EMS). EMS reported oxygen saturation in the mid-80% range, tachypnea, and a systolic blood pressure of 244 mmHg. He was placed on continuous positive airway pressure with improvement in oxygenation and was subsequently transitioned to 4 L nasal cannula oxygen. Upon arrival to the emergency department, vital signs were notable for blood pressure of 135/68 mmHg, heart rate of 130 beats/minute, respiratory rate of 28 breaths/minute, and oxygen saturation of 95% on a 4 L nasal cannula. Physical examination was remarkable for tachycardia with regular rhythm and no murmurs, bilateral wheezing and diffuse crackles on lung auscultation, and significant abdominal distention with diffuse tenderness and guarding without rigidity. Initial laboratory studies were notable for leukocytosis at 13.9 × 10³/µL, slightly elevated creatinine at 1.3 mg/dL (baseline previously recorded to be 0.9-1.1 mg/dL), lactic acid at 2.8 mmol/L, and elevated high-sensitivity troponin at 234 pg/mL, with a repeat level of 197 pg/mL one hour later (delta: −37 pg/mL), suggesting demand-mediated ischemia. Nasopharyngeal polymerase chain reaction testing was positive for RSV. Chest radiograph demonstrated patchy left basilar and right mid-lung airspace opacities. Computed tomography (CT) of the abdomen and pelvis without intravenous (IV) contrast revealed a perforated sigmoid diverticulitis with small foci of pneumoperitoneum, ill-defined fluid in the left lower quadrant without a discrete walled-off collection, and a small amount of free fluid, determined to be likely reactive in nature (Figures 1, 2).

**Figure 1:**
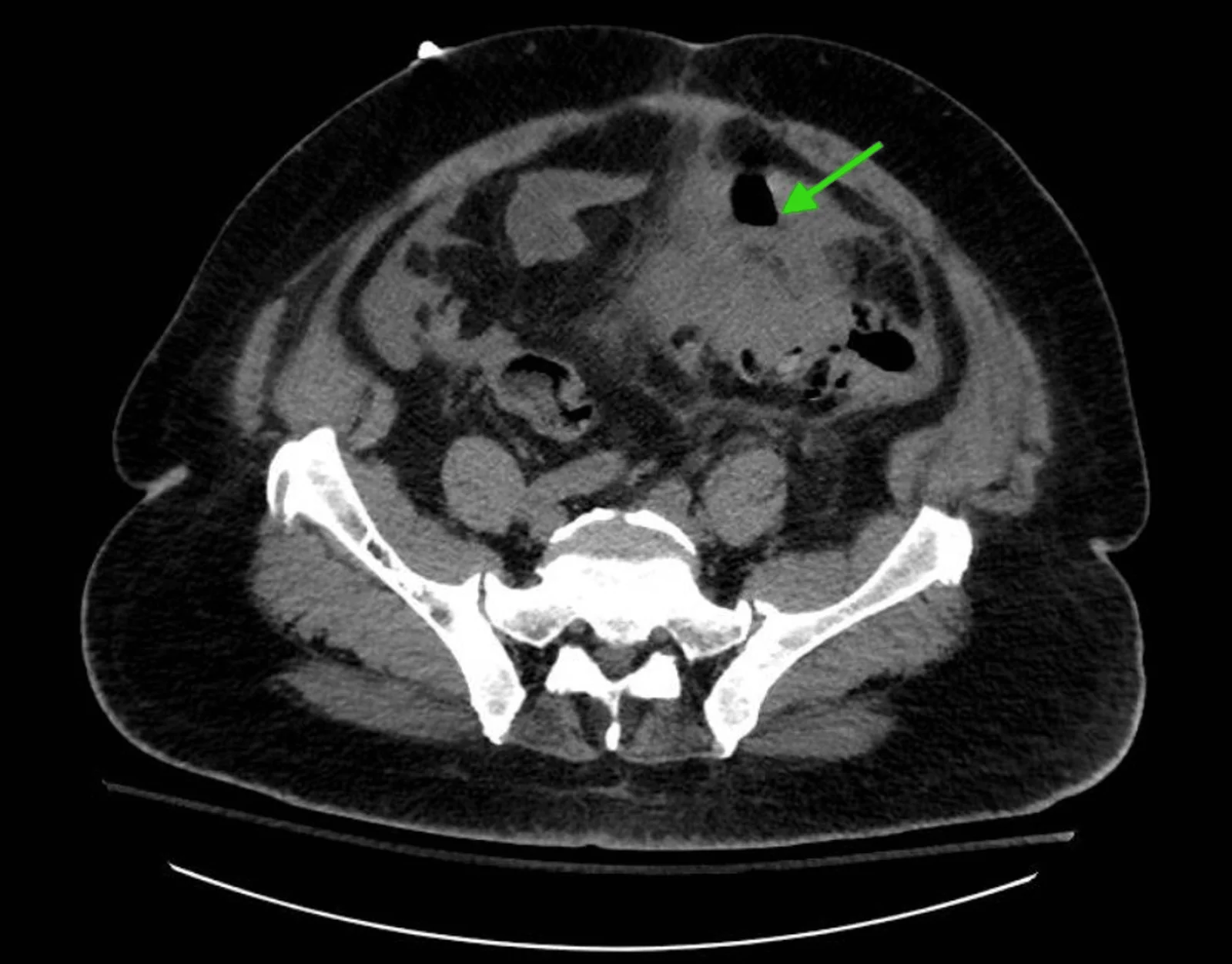
Transverse view of computed tomography of the abdomen/pelvis without intravenous contrast demonstrating ill-defined fluid in the left lower quadrant with perforated sigmoid diverticulitis (green arrow).

**Figure 2:**
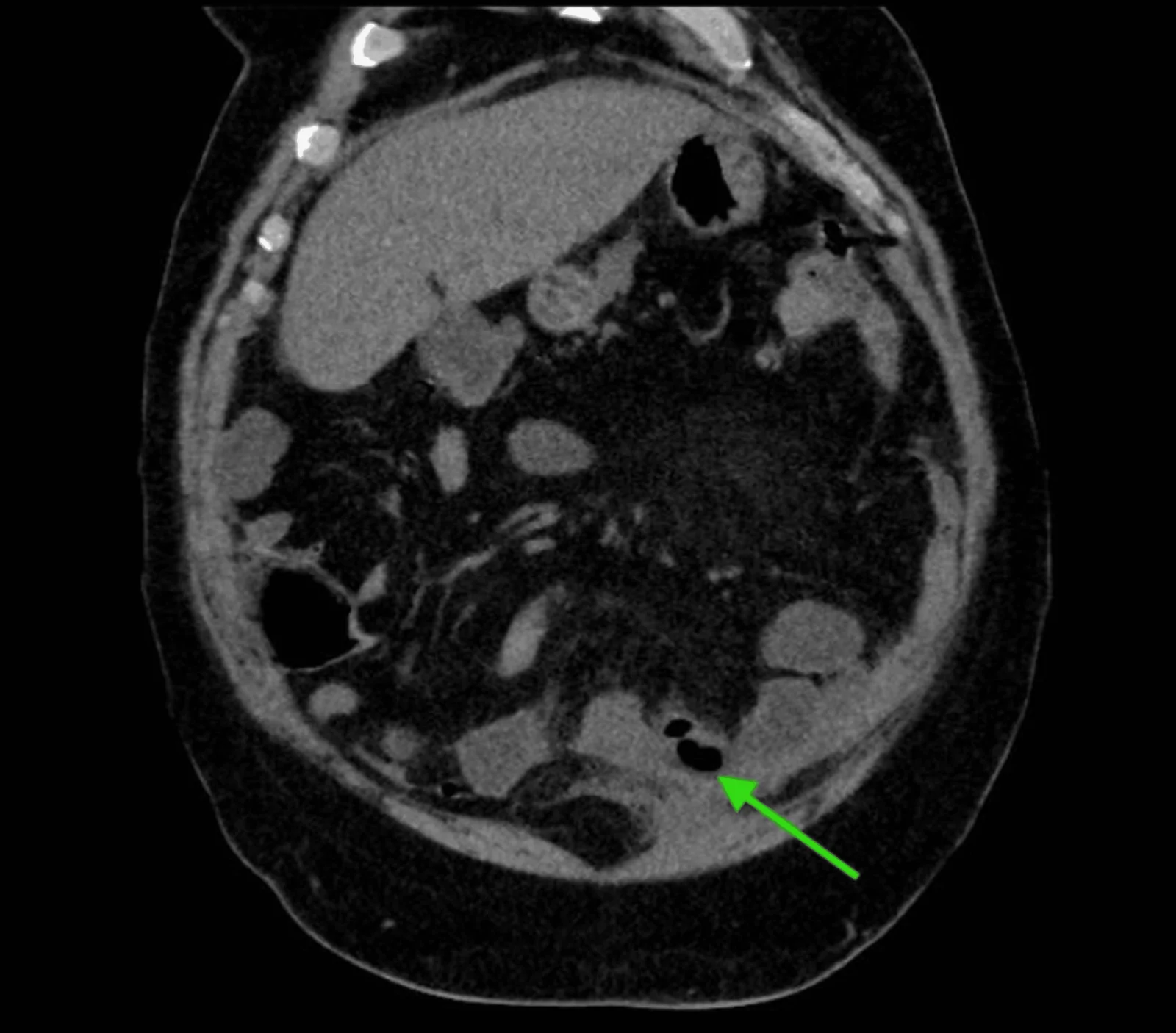
Coronal view of computed tomography of the abdomen/pelvis without intravenous contrast demonstrating ill-defined fluid in the left lower quadrant with perforated sigmoid diverticulitis (green arrow).

The patient was diagnosed with sepsis based on the Systemic Inflammatory Response Syndrome criteria, and the suspected source of infection and blood cultures were obtained. He was started on IV piperacillin-tazobactam with IV normal saline for fluid resuscitation. Surgical consultation determined that the patient was at high perioperative risk, and conservative management was recommended. Blood cultures subsequently grew *Bacteroides thetaiotaomicron*. The patient initially demonstrated symptomatic improvement with antibiotic therapy. However, five days after admission, repeat CT imaging of the abdomen revealed two organized fluid collections in the left abdomen concerning for abscess formation (Figure 3).

**Figure 3:**
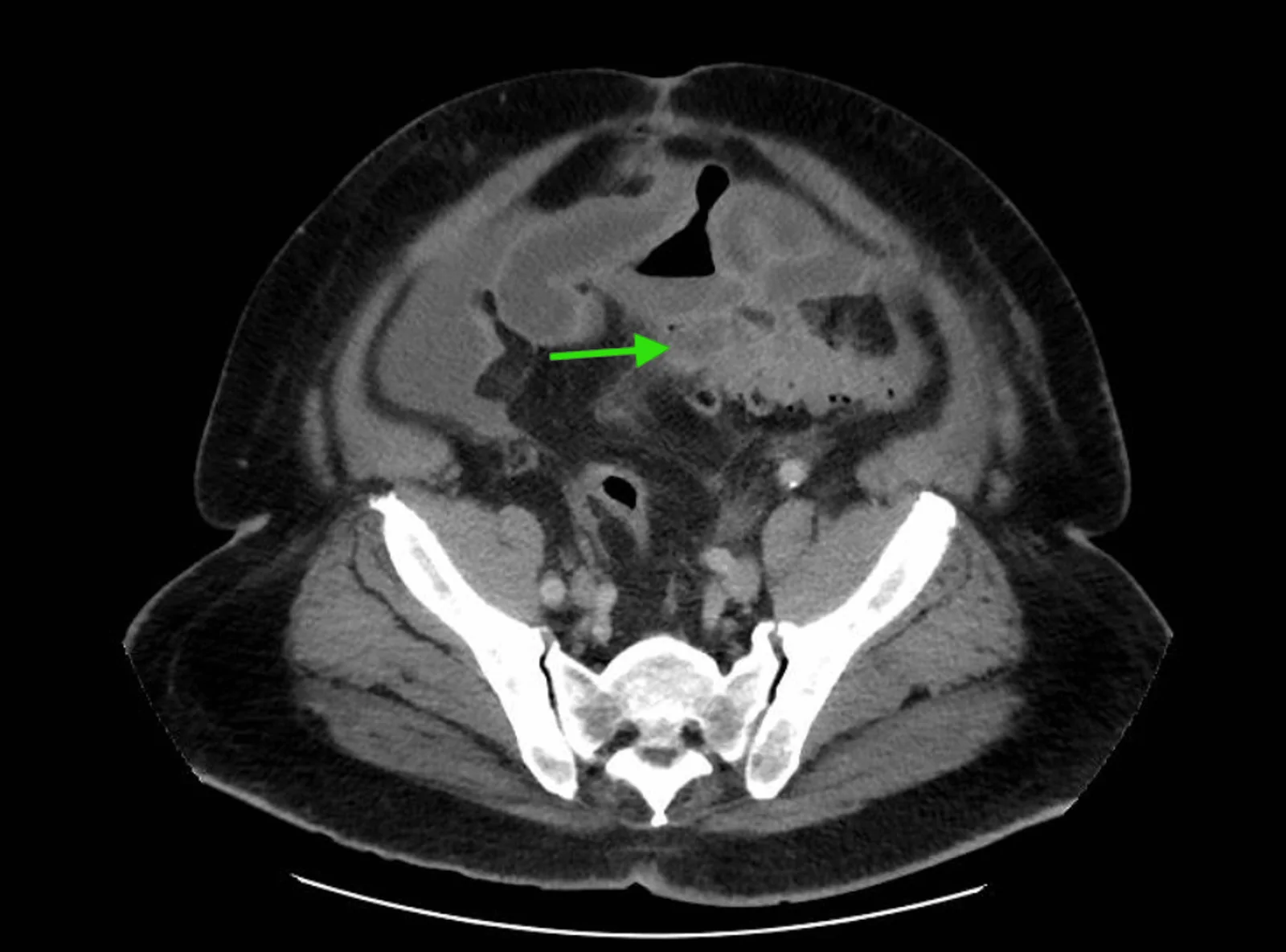
Transverse view of computed tomography of the abdomen/pelvis with intravenous contrast completed five days after admission. Abscess formation can be seen (green arrow).

Interventional radiology performed CT-guided percutaneous drainage with placement of a percutaneous drain, and fluid samples were sent for culture. Additionally, a peripherally inserted central catheter was placed for initiation of total parenteral nutrition (TPN). At this point, the patient’s respiratory symptoms had largely resolved, and he no longer required supplemental oxygen. During hospitalization, serial complete blood counts demonstrated progressive anemia without overt signs of bleeding, with hemoglobin declining to 7 g/dL, prompting transfusion of one unit of packed red blood cells. Follow-up CT obtained five days after the initial drainage procedure demonstrated improvement in the previously drained collections but revealed multiple new abscesses in the abdomen and pelvis, the largest measuring 8.7 cm in size (Figure 4).

**Figure 4:**
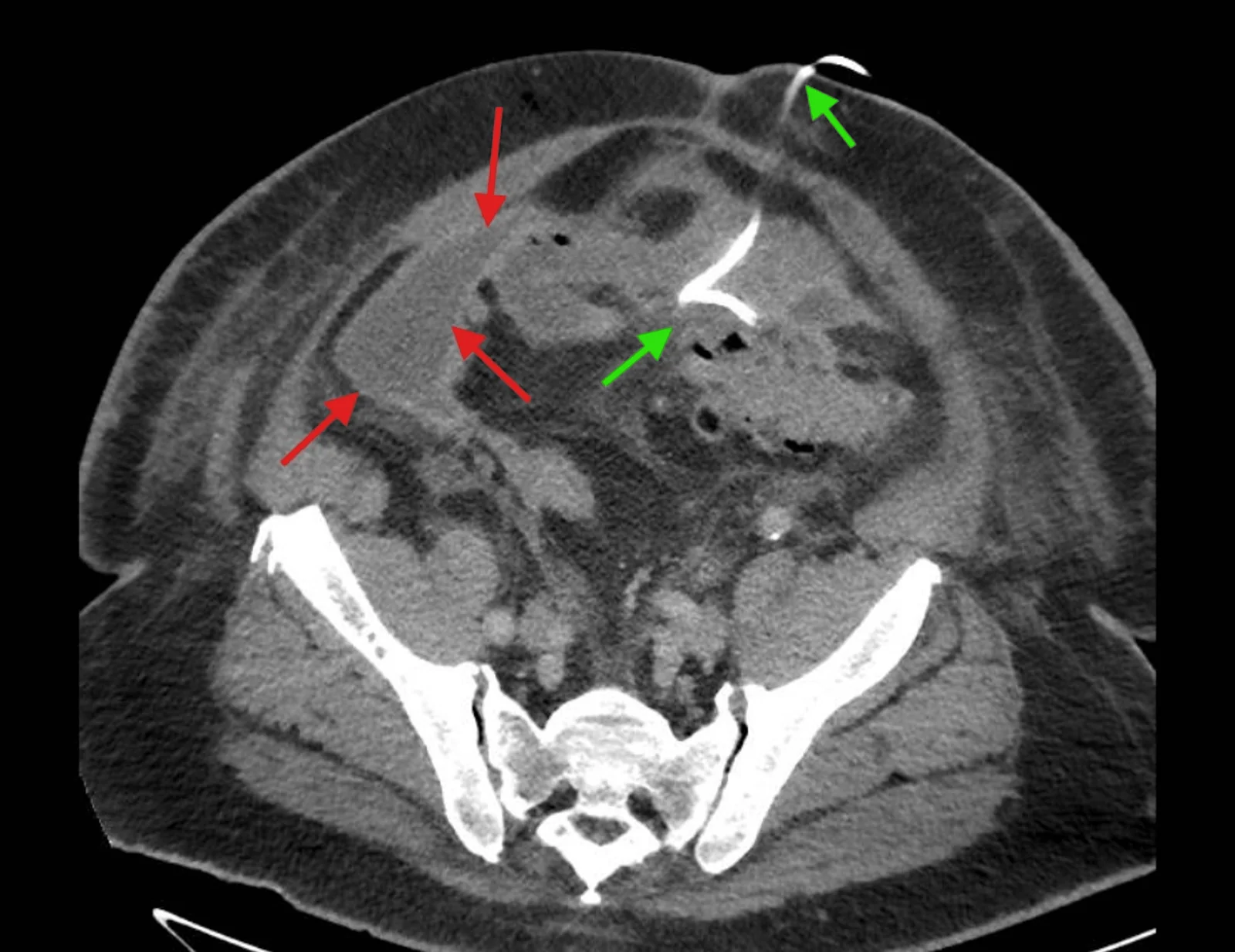
Follow up computed tomography five days after percutaneous drain placement. Percutaneous drain can be visualized where initial abscess was located (green arrows) and a new 8.7 cm fluid collection can be seen in the same plane (red arrows).

A second CT-guided percutaneous drain was subsequently placed into the largest fluid collection. Cultures from the initial abscess drainage grew *Enterobacter cloacae* and *Pseudomonas aeruginosa*; therefore, antibiotic therapy was adjusted to IV cefepime and metronidazole, due to the potential for AmpC beta-lactamase production by *Enterobacter* species. The patient continued to improve clinically with antibiotic therapy, percutaneous drainage, and nutritional support. Over time, he was able to transition from TPN to a clear liquid diet and eventually to a low-residue diet without complications. The original drain had minimal drainage at the end of this hospital course and was subsequently removed, with retention of the second drain for outpatient management and follow-up. The patient was ultimately discharged with planned outpatient follow-up; however, he was lost to follow-up with the hospital team.

## Discussion

This case describes concurrent RSV infection and perforated sigmoid diverticulitis with subsequent intra-abdominal abscess formation in an older adult with a known history of diverticulosis. It is important to acknowledge that a perforated diverticulitis alone represents a well-recognized cause of bowel perforation and likely accounts for the primary pathology in this patient. Chart review revealed that the patient had previously documented diverticulosis on imaging in 2022, which represents a known risk factor for diverticulitis and subsequent perforation. To our knowledge, there is currently no established evidence demonstrating a direct causal relationship between RSV infection and gastrointestinal perforation in adults, and this report does not intend to suggest that RSV was the definitive cause of this patient’s perforation.

It is worth reporting, however, that prior studies have highlighted that RSV infection may be associated with gastrointestinal pathology in pediatric populations. Cases of necrotizing enterocolitis have been reported in term and late-term infants with concurrent RSV infection [6]. Though the exact mechanism of this sequela in children remains unclear, it may involve a dysregulated proinflammatory response, causing alterations in intestinal blood flow and possibly contributing to compromised barriers against bacterial translocation in these children. Necrotizing enterocolitis is a known complication of preterm birth; however, in the aforementioned study, the patients examined were of both term and late-term births [5,6].

Another speculated mechanism for injury is RSV-induced hypoxia and hypoperfusion leading to intestinal mucosal injury [7]. Given the onset of respiratory symptoms for our patient and the initial presentation with hypoxia requiring non-invasive ventilatory support, it is possible that transient hypoxic episodes may have induced damage and disrupted the integrity of the intestinal mucosal barrier. The timing of the acute RSV infection and the development of severe gastrointestinal complications raises the possibility that RSV-associated systemic inflammation or hypoxia may have contributed to the mucosal compromise. Patients with multiple comorbidities, including cardiovascular disease and diabetes mellitus, may be particularly susceptible to such systemic effects. However, as stated, this is hypothesized and is not definitively causal in nature. Our patient, as mentioned, had several comorbidities that may have contributed to his presentation, such as known diverticulosis, type 2 diabetes mellitus, and recent NSTEMI. Additionally, it is possible the RSV contributed to his condition by purely mechanical means, such as increases in intra-abdominal pressure with coughing secondary to RSV; however, this is purely speculative. Further research is required to better understand whether RSV infection may predispose susceptible adult patients to gastrointestinal complications or exacerbate underlying gastrointestinal pathology.

## Conclusions

This case report describes a concurrent RSV infection and perforated sigmoid diverticulitis complicated by multiple intra-abdominal abscesses requiring percutaneous drainage in an older adult. Although diverticulitis likely represents the primary etiology, RSV-associated systemic inflammation, hypoxia, or increases in intra-abdominal pressure secondary to coughing may have contributed to gastrointestinal mucosal injury in this patient. Reports describing gastrointestinal complications associated with RSV in adults remain limited. Further investigation is needed to determine whether RSV infection may predispose susceptible individuals to gastrointestinal pathology.
